# F11-Mediated Inhibition of RhoA Signalling Enhances the Spread of Vaccinia Virus In Vitro and In Vivo in an Intranasal Mouse Model of Infection

**DOI:** 10.1371/journal.pone.0008506

**Published:** 2009-12-30

**Authors:** João V. Cordeiro, Susana Guerra, Yoshiki Arakawa, Mark P. Dodding, Mariano Esteban, Michael Way

**Affiliations:** 1 Cell Motility Laboratory, London Research Institute, London, United Kingdom; 2 Department of Molecular and Cellular Biology, Centro Nacional de Biotecnología, Madrid, Spain; 3 Department of Preventive Medicine and Public Health, Universidad Autónoma, Madrid, Spain; 4 Department of Neurosurgery, Kyoto University Graduate School of Medicine, Kyoto, Japan; University of Birmingham, United Kingdom

## Abstract

The cortical actin cytoskeleton beneath the plasma membrane represents a physical barrier that vaccinia virus has to overcome during its exit from an infected cell. Previous observations using overexpression and pharmacological approaches suggest that vaccinia enhances its release by modulating the cortical actin cytoskeleton by inhibiting RhoA signalling using the viral protein F11. We have now examined the role of F11 and its ability to interact with RhoA to inhibit its downstream signalling in the spread of vaccinia infection both in vitro and in vivo. Live cell imaging over 48 hours reveals that loss of F11 or its ability to bind RhoA dramatically reduces the rate of cell-to-cell spread of the virus in a cell monolayer. Cells infected with the ΔF11L virus also maintained their cell-to-cell contacts, and did not undergo virus-induced motility as observed during wild-type infections. The ΔF11L virus is also attenuated in intranasal mouse models of infection, as it is impaired in its ability to spread from the initial sites of infection to the lungs and spleen. Loss of the ability of F11 to bind RhoA also reduces viral spread in vivo. Our results clearly establish that viral-mediated inibition of RhoA signalling can enhance the spread of infection not only in cell monolayers, but also in vivo.

## Introduction

Vaccinia virus, the prototypic and most characterized member of the *orthopoxvirus* genus of the *poxvirdae* is a large double stranded DNA virus that replicates in the cytoplasm of its infected host cell [1], [2], [3]. Replication and viral particle assembly, which involves a complex series events that are still not fully understood, occurs within viral factories localized at or near the microtubule-organizing center of the cell [1], [2], [3]. Replication initially results in the formation of intracellular mature virions (IMV) around 5–6 hours post infection, although this can vary depending on the cell type. IMV are infectious but are principally only released from the infected cells when they undergo lysis. During the vaccinia infection cycle a proportion of IMV can also become wrapped by a membrane cisternae derived from the Trans-Golgi network or endosomal compartments that contain a subset of integral viral membrane proteins [3], [4]. This envelopment results in the formation of intra-cellular enveloped virions (IEV), which are capable of undergoing kinesin-1 dependent microtubule transport from their peri-nuclear site of assembly to the cell periphery [5], [6], [7], [8], [9], [10].

Upon reaching the cell periphery IEV switch transport systems and undergo actin-dependent movements in the cell cortex [11]. These viral movements, which have an average speed of 0.35 µm sec^−1^, are suggestive of a myosin dependent transport step through the cortical actin prior to fusion with the plasma membrane [11]. Fusion of the IEV with the plasma membrane results in the formation of the extra-cellular virions, a large proportion of which, remain associated with the outside of the cell [3], [4]. These cell associated virus particles are able to locally activate Src and Abl family kinases to induce phosphorylation of an integral IEV membrane protein A36 [12], [13], [14], [15], which is localized in the plasma membrane beneath the virus particle [4], [16], [17]. Phosphorylation of A36 leads to the recruitment of a signalling complex, consisting of Grb2, Nck, WIP (WASP interacting protein) and N-WASP that is able to locally activate the Arp2/3 complex to stimulate polymerization of actin beneath the extracellular virus [12], [18], [19], [20], [21], [22]. The polymerization of actin beneath the virus particle provides a driving force that helps to enhance the cell-to-cell spread of the virus [3], [6], [9], [23], [24].

In the absence of cell lysis, newly assembled vaccinia virions are largely only released from the infected cell, when IEV fuse with the plasma membrane. However, to reach the plasma membrane the IEV first have to traverse the cortical actin cytoskeleton, which consists of an extremely dense arrangement of actin filaments beneath the plasma membrane. This dense actin cortex represents a significant physical barrier to IEV, given their large size [25]. Vesicles and secretory granules also face a similar problem during exocytosis [26], [27]. A variety of studies have shown that the remodelling of cortical actin as well as active actin polymerization plays an essential role in facilitating exocytosis [26], [27]. It is therefore not surprising given their size, that the release of IEV from infected cells is also dependent on the assembly and organization of the cortical actin cytoskeleton [11]. In addition, vaccinia appears to enhance its release from infected cells in culture by modulating the cortical actin by inhibiting RhoA signalling to mDia, a key regulator of actin polymerization [11]. The virus achieves this by encoding F11, a protein that mimicks ROCK to interact with RhoA to inhibit its downstream signalling [28], [29]. F11-mediated inhibition of RhoA signalling is also responsible for stimulating virus-induced cell migration, which may also help to enhance the spread of infection [28], [30]. Our previous observations on the role of F11 in promoting viral release are based on the effects of over expressing dominant negative and activated RhoA and mDia clones coupled with pharmalogical approaches to modulate RhoA-mDia signalling and the actin cytoskeleton. To extend these observations and directly investigate whether that F11-mediated inhibition of RhoA signalling promotes viral release and spread we have generated recombinant viruses lacking F11 or expressing an F11 mutant (F11-VK), which is deficient in binding RhoA [28]. We found that loss of F11 or its ability to bind RhoA significantly reduces the release of infectious virus from infected cells. Live cell imaging reveals that there was a corresponding reduction in the rate of the spread of infection in cell monolayers in culture. We also found that the ability of F11 to bind RhoA also enhances viral spread *in vivo* in an intranasal mouse model of infection.

## Results

### F11 Binds RhoA to Inhibit Its Signalling during Infection

**Figure 1:**
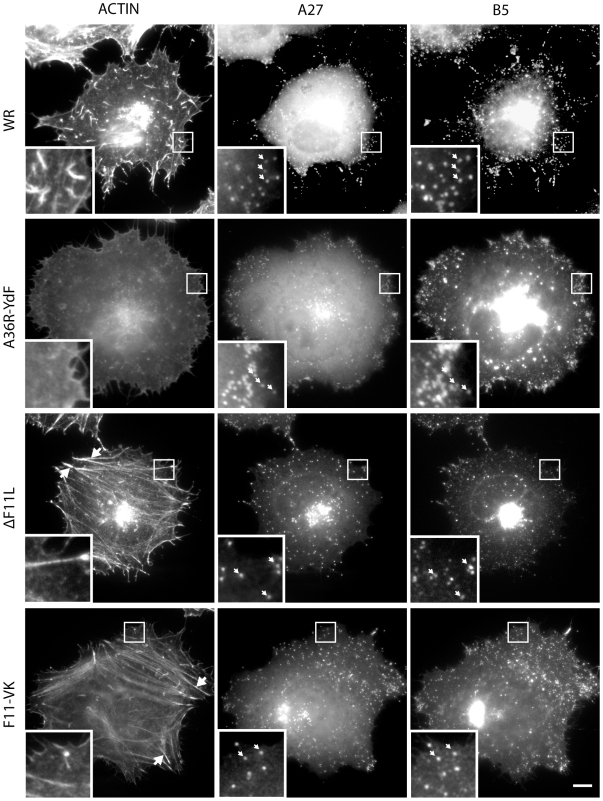
F11 promotes loss of actin stress fibres during infection. (**A**) Representative images of infected HeLa cells stained with the viral markers A27 and B5 reveals that loss of F11or its ability to bind RhoA (F11-VK) does not affect virus particle assembly (white arrow heads in inserts). The presence of actin stress fibres (large white arrows) in ΔF11L and F11-VK but not WR or A36R-YdF infected cells is indicative of active RhoA signalling. Scale bar = 10 µm.

**Figure 2:**
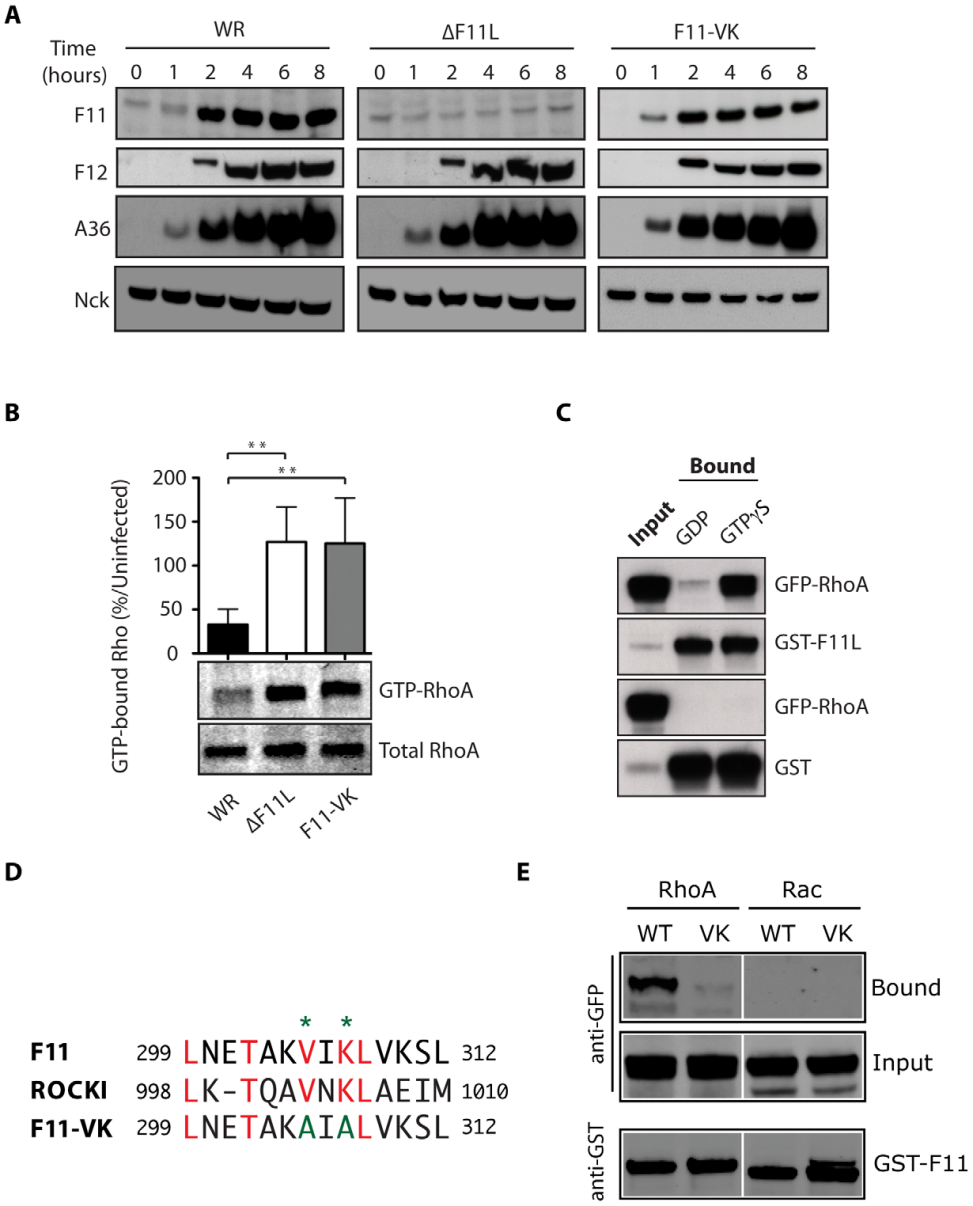
F11 binds GTP-bound RhoA to inhibit its signalling during infection. (**A**) Immunoblot analysis reveals that F11 is detected as early as 2 hours post infection in WR and F11-VK but not ΔF11L infected cells. The kinetics of A36 and F12 expression are unaffected by the loss of F11 and Nck represents a loading control. (**B**) Quantitative immunoblot analysis of Rho activation Rhotekin pull down assays reveals that WR but not ΔF11L or F11-VK reduces the level of GTP-bound RhoA at 8 hours post infection as compared to non-infected controls. A representative immunoblot of the level of GTP-bound and total RhoA is shown. Error bars represent standard error of the mean (SEM) from 4 independent experiments and * = P<0.05. (**C**) Immunoblot analysis of glutathione resin pull down assays from vaccinia virus infected cell extracts, reveals that GST-tagged F11 interacts with GTPγS (GTP) but not GDP bound RhoA. (**D**) Sequence alignment of F11 and the RhoA binding motif in ROCK1. Identical residues are shown in red and the positions of valine 305 and lysine 307 in F11 that are mutated to alanine in the F11-VK mutant are indicated. (**E**) Immunoblot analysis of glutathione resin pull down assays reveals that GST-tagged F11 but not F11-VK interacts with GFP-RhoA. Neither protein interacts with GFP-Rac. (**D**).

Consistent with previous observations, immunofluorescence analysis reveals that infection with the Western Reserve (WR) strain of vaccinia results in the loss of actin stress fibres in addition to the formation of numerous virus tipped actin tails (Fig. 1) [28], [31]. The loss of actin stress fibres is not due to the formation of virus tipped actin tails as cells infected with the WR-A36R-YdF virus, which is deficient in actin tail formation [7], also loose their actin stress fibres (Fig. 1). In contrast, HeLa cells infected with the recombinant ΔF11L virus, which does not express F11 (Fig. 2A), have significantly more actin stress fibres than those infected with WR or A36R-YdF at 8 hours post-infection (Fig. 1). The presence of more actin stress fibres suggests that the level of RhoA signalling is higher in ΔF11L as compared to WR infected cells. Quantitative western blot analysis of Rhotekin-pull-down assays on infected cell extracts confirmed that the level of GTP-bound RhoA is significantly higher in ΔF11L than WR infected cells at 8 hours post infection (Fig. 2B). These results using a recombinant virus is consistent with our previous observations using RNAi mediated ablation of F11 expression during infection [28], [29]. Consistent with its ability to inhibit RhoA signalling by mimicking the Rho binding motif of ROCK [28], we found that F11 interacted specifically with GTP (GTPγS) but not GDP bound GFP-RhoA (Fig. 2C). This contrasts our previous observations which showed F11 can bind both V14 (active GTP mimick) and N19 (inactive GDP mimick) RhoA mutants [28].

To confirm that the loss of actin stress fibres observed during WR infection are due to the loss of a direct interaction between F11 and RhoA rather than the consequence of indirect signalling effects, we generated a recombinant virus expressing a mutant version of the protein (F11-VK), which is unable to bind RhoA (Fig. 2D, E) [28]. Western blot analysis of infected cells reveals that F11-VK is expressed at a similar time and level as the wild type protein (Fig. 2A). Infection with the F11-VK virus, however, did not result in a reduction in the level of GTP-bound RhoA at 8 hours post infection as seen with WR (Fig. 2B). Moreover, the level of GTP-bound RhoA in F11-VK infected cells is similar to that observed during ΔF11L infections (Fig. 2B). Consistent with this, we observed that F11-VK infected cells also had prominient actin stress fibres at 8 hours post infection (Fig. 1). Our observations demonstrate that the direct binding of F11 to GTP bound RhoA and subsequent inhibition of its down stream signalling is required for the remodelling of the actin cytoskeleton observed during vaccinia infection.

### F11-Mediated Loss of RhoA Signalling Enhances the Cell-to-Cell Spread of Vaccinia

**Figure 3:**
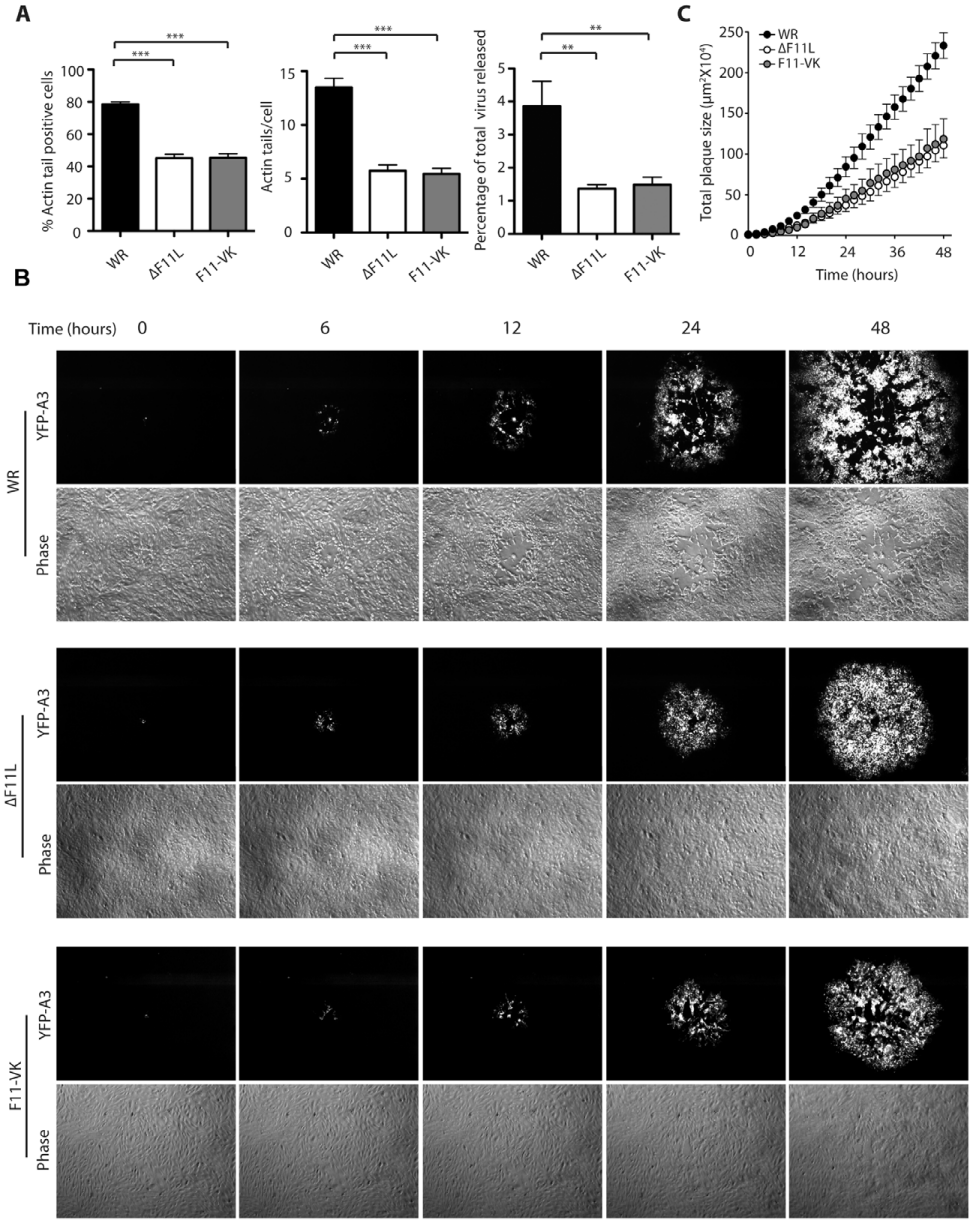
F11 enhances the cell-to-cell spread of vaccinia virus. (**A**) Quantitative analysis reveals that ΔF11L or F11-VK produce significantly less actin tails than WR at 8 hours post infection. A lack of inhibition of RhoA signalling also results in a ∼2.5 fold reduction in the % of infectious virus released into the media from ΔF11L or F11-VK as compared to WR infected cells. Error bars represent SEM from 4 independent experiments and ** indicates P<0.01 and *** indicates P<0.001. (**B**) Phase contrast and fluorescence movie stills of a developing plaque at the indicated time reveals that WR spreads faster in a monolayer of BS-C-1 cells than the ΔF11L or F11-VK viruses (Movies S1, S2, S3). The central clearing seen in WR and F11-VK plaques is due to a loss of cell-cell adhesion and viral induced cell migration. (**C**) Quantitative analysis of the rate of spread of the indicated virus during 48 hours. Error bars indicate SEM from 6 independent plaques.

We observed that the ΔF11L and F11-VK viruses were still capable of inducing the formation of actin tails. However, it was noticeable that cells infected with the ΔF11L and F11-VK viruses had significantly fewer actin tails than those infected with WR (Figs. 1, 3A). This similar reduction in the number of actin tails could be due to defects in virus replication and assembly. However, F11 is not required for virus replication and assembly [30], [32]. An alternative explanation for the reduction in number of actin tails is that the inability of the ΔF11L and F11-VK viruses to down regulate RhoA signalling impairs the ability of IEV particles to reach and fuse with the plasma membrane. In agreement with this, we found that there is a significant reduction in the percentage of infectious virus particles released into the media from cells infected ΔF11L and F11-VK viruses as compared to WR (Fig. 3A). These data, which agree with our previous observations using RNAi and over expression approaches in WR infected cells[11], suggest that F11-mediated inhibition of RhoA is likely to play an important role in the cell-to-cell spread of vaccinia.

To directly examine if this is the case, we imaged the spread of WR, ΔF11L and F11-VK viruses expressing YFP-A3, an abundant viral core protein, in confluent monolayers of BS-C-1 cells during plaque formation. The cell monolayer was imaged for 48 hours immediately following the detection of YFP-A3 expression in the perinuclear region of a single infected cell ∼8 hours post infection (Fig. 3B, Movies S1, S2, S3). To quantify the rate of cell-to-cell spread of each virus, we measured the area of YFP-A3 positive cells over time. In all three cases, after an initial delay, corresponding to ∼10 hours there was a linear increase in the size of the developing plaque (Fig. 3C). The loss of F11 or its ability to interact with RhoA both resulted in a similar dramatic reduction in the rate at which the virus was able to spread as compared to WR (Fig. 3B, C, Movies S1, S2, S3).

**Figure 4:**
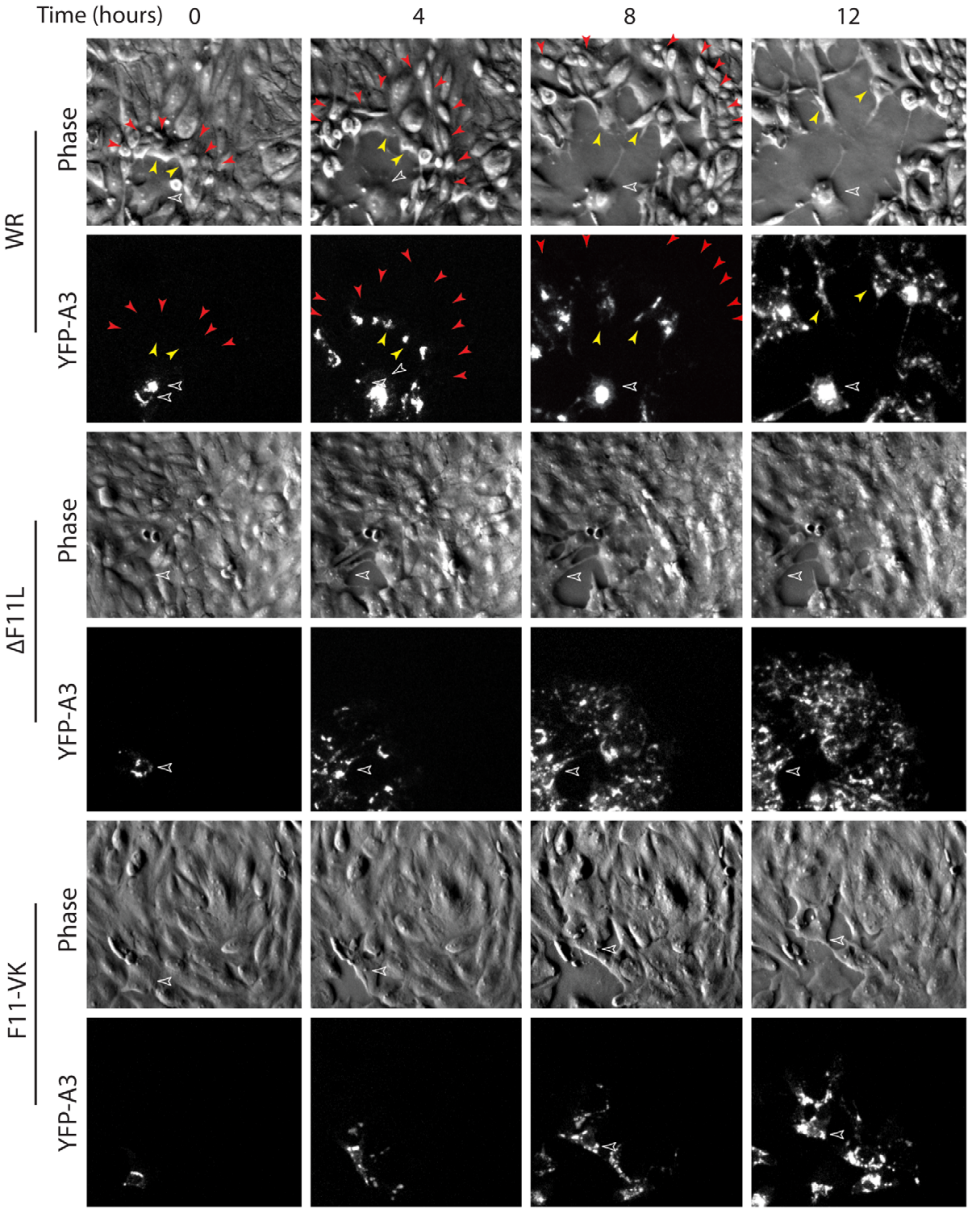
F11 promotes migration of infected cells. (**A**) Phase contrast and fluorescence movie stills at the indicated time of the leading edge of a developing plaque induced by WR expressing YFP-A3L. Yellow arrowheads highlight infected cells migrating away from the initial infected cell (open white arrowhead). Red arrowheads indicate cells that contract at the plaque front prior to moving (Movies S4, S5, S6).

**Figure 5:**
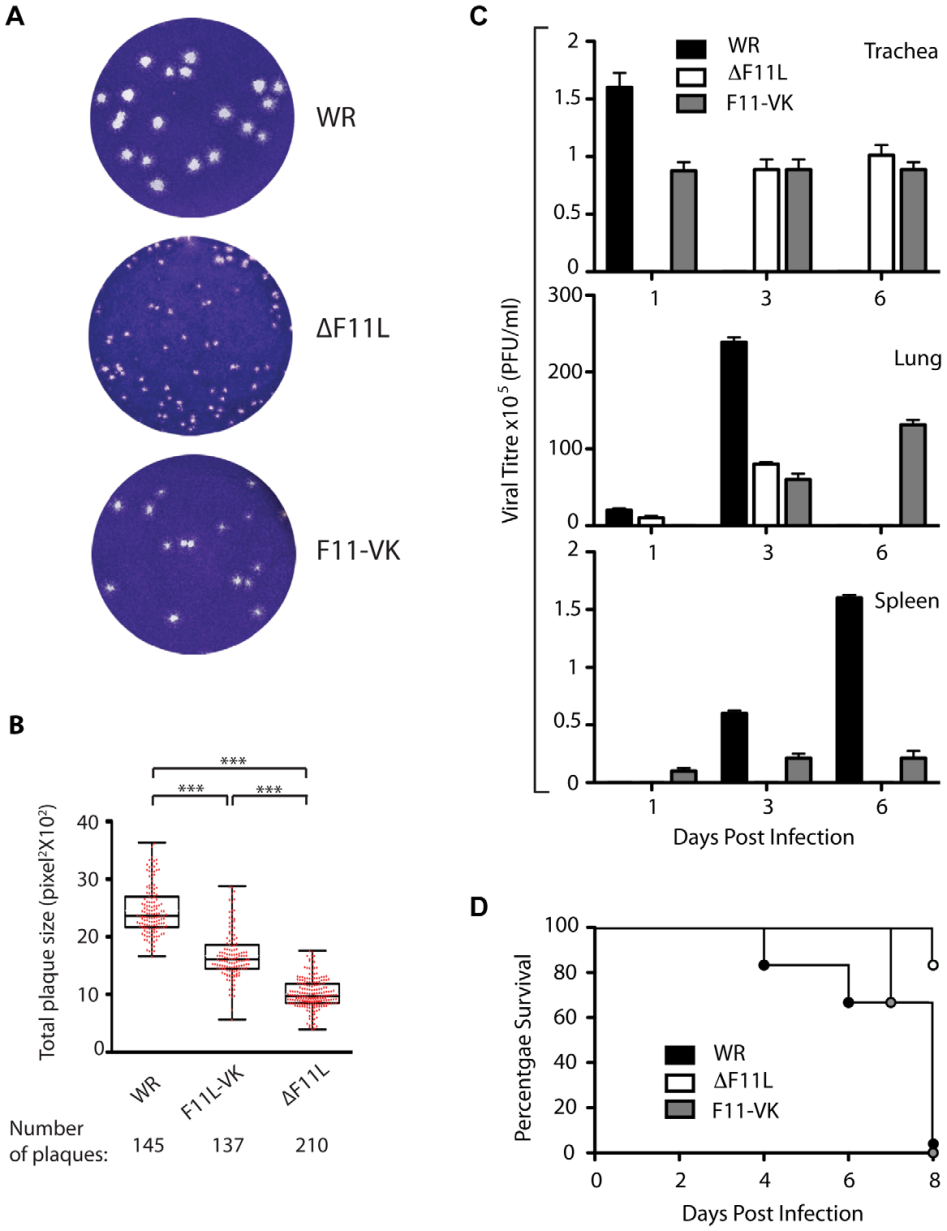
Loss of F11 limits spread of infection in vitro and in vivo. (**A**) Representative images of plaques formed by WR, ΔF11L and F11-VK at 4 days post infection. (**B**) Quantitative analysis of the size of plaques formed by WR, ΔF11L and F11-VK are shown as Box-and-whisker plots overlaid with the data points (red). The boxes represent 25th and 75th percentile of the data, the line bisecting the box represents the median and *** indicates P<0.001. (**C**) Quantitative analysis of viral titres in the trachea, lungs and spleens of WR (black bars), ΔF11L (white bars) or F11-VK (grey bars) infected mice at 1, 3 and 6 days post inoculation. Error bars indicate SEM from five mice and the minimum limit of detection is 100 pfu/g tissue. (**D**) Survival rates of C57/BL6 mice infected intra-nasally with 5×10^6^ pfu of WR, ΔF11L and F11-VK viruses (n = 6).

During the spread of WR the integrity of the cell monolayer was disrupted due to the loss of cell-cell adhesion and migration of cells away from the centre of the plaque (Figs. 3B, 4, Movies S4, S5, S6). It was also noticeable that during the spread of WR there is a prominent “wave front” in the phase image near the edge of the developing plaque (Fig. 3B, Movie S1). Closer examination reveals that this “wave front” corresponds to a combination of cells undergoing migration and contracting ∼2–3 hours before YFP-A3 is detected (Fig. 4, Movie S4). In contrast, although there is some loss of cell-cell adhesion at the initial site of infection, ΔF11L infected cells did not contract or undergo any appreciable cell migration (Figs. 3B, 4, Movies S2, S5). F11-VK infected cells also did not contract when infected (Figs. 3B and 4, Movies S3, S6). They did however loose some cell-cell adhesion and migrate, albeit to a much lesser degree than WR infected cells (Figs. 3B and 4, Movies S3, S6). The differences in cell adhesion and migration between ΔF11L and F11-VK infected cells do appear to affect viral spread as plaques formed by the F11-VK virus were larger than those formed ΔF11L at four days post infection (Fig. 5A, B). Plaques formed F11-VK were however still significantly smaller than that observed for WR at four days post infection (Fig. 5A, B).

### F11-Mediated Inhibition of RhoA Enhances Spread of Vaccinia In Vivo

Our observations in confluent cell monolayers in culture indicate that F11 and its abililty to bind RhoA to inhibit its downstream signalling enhances the cell-to-cell spread of infection. To explore whether F11-mediated inhibition of RhoA signalling actually contributes to the pathogenesis of vaccinia virus *in vivo*, we infected C57BL6 mice intranasally with 5×10^6^ plaque-forming units of purified WR, ΔF11L or F11-VK viruses. Intranasal innoculation of WR results in an acute infection of the lung followed by subsequent spread of extra-cellular virus to the visceral organs of the mouse [33].

To quantify the extent of virus spread *in vivo* we performed plaque assays on trachea, lung and spleen extracts 1, 3 and 6 days after intranasal inoculation. We found that high titres of WR were detected in the trachea of animals one day post inoculation (Fig. 5C). By day 3, WR had progressed to the lungs and subsequently to the spleen by day 6. In contrast, the ΔF11L virus was mainly detected in the trachea at 3 days post inoculation and similar levels of the virus still persisted in the tissue at day 6 (Fig. 5C). The ΔF11L virus was also impaired in its ability to spread from the trachea, as low titres were detected in the lungs on day 3 and it did not reach the spleen. Moreover, the titre of ΔF11L in the lungs on day 3 was significantly lower compared to that of WR. The F11-VK virus gave an intermediate phenotype between WR and ΔF11L. F11-VK was detected in the trachea and lungs at the same time as WR albeit at reduced titres (Fig. 5C). However, it also persisted in the trachea as observed with ΔF11L. In contrast to WR, F11-VK was also still present in the lungs at 6 days post infection but was impaired in its ability to reach the spleen.

Consistent with its ability to spread and replicate, mice infected with WR started dying at 4 days post-inoculation and had all died by day 8 (Fig. 5D). In contrast, most of the ΔF11L infected mice still survived at 8 days post infection (Fig. 5D). Animals infected with the F11-VK virus initially survived longer than those infected with WR but were also all dead by day 8. Taken together, our analysis reveals that F11-mediated inhibition of RhoA signalling acts to enhance the spread of infection not only *in vitro* but also *in vivo*.

## Discussion

During recent years it has become clear from studies performed in cell culture that viruses take advantage of the cytoskeleton of their hosts to help faciliate their entry, replication and subsequent spread [34], [35], [36], [37], [38]. This viral mediated exploitation of the cytoskeleton often also involves manipulation of signalling pathways involving Src and Abl kinases as well as RhoGTPases [39], [40]. In contrast to the situation in infected cells in culture, we still understand very little concerning the role of the cytoskeleton and its manipulation during viral spread and pathogenesis *in vivo*.

In the case of vaccinia virus, Gleevec mediated inhibition of Abl and Arg tyrosine kinases reduces viral spread *in vivo* and also protects mice from a lethal intranasal innoculation [14]. These *in vivo* observations are consistent with data obtained from cells in culture demonstrating that Abl family kinases are involved in vaccinia actin tail formation and promoting the release of virus from the surface of infected cells [14], [15]. More recently, we found that F11-mediated down regulation of RhoA signalling also promotes the release of virus from infected cells in culture [11]. This conclusion was based on the effects of over-expression of dominant negative and active RhoA and mDia clones combined with pharmacological inhibition of RhoA signalling and actin cytoskeleton dynamics. In our current study, we directly examined whether inhibition of RhoA signalling promotes viral release and spread using recombinant viruses lacking the F11L gene or expressing an F11 mutant that is deficient in RhoA binding. Our data now clearly demonstrates using an intranasal mouse model of infection that F11-mediated inhibition of RhoA signalling enhances the spread of vaccinia infection not only in cell monolayers but also *in vivo*.

Consistent with previous publications using recombinant viruses with nonsense mutations in the F11L gene [30], [32], we found that deletion of the majority of the F11L gene and loss of F11 expression did not affect viral morphogenesis. The loss of F11 or its ability to bind RhoA did however result in significantly higher levels of GTP bound RhoA at 8 hours post infection, consistent with the presence of actin stress fibres in ΔF11L or F11-VK as compared to WR or WR-A36R-YdF infected cells. We also found that the increased RhoA signalling resulted in a significant reduction in the number of actin tails and amount of virus released into the culture medium. This reduction must at least in part be responsible for the smaller plaques formed by the ΔF11L and F11-VK viruses, as the ability to induce actin tails is know to promote the formation of larger plaques and enhance viral spread [3], [9].

Although plaque size gives a measure of the efficiency of cell-to-cell spread it provides no information concerning the dynamics of the process as it represents a single fixed time point. Given this we imaged viral spread in live cells during plaque formation to assess more directly how F11-mediated inhibition of RhoA signalling enhances the spread of infection. We found that F11 promotes the spread of infection not only by enhancing viral release but by also stimulating the migration of infected cells after their loss of cell-cell adhesion. Our findings support and extend previous suggestions and observations in isolated cells [11], [28], [30]. The loss of cell-cell adhesion during WR infection is also consistent with the known role of RhoA signalling in establishing and maintaining cell-cell contacts [41], [42].

It was noticeable that cells within the monolayer contracted, even though they are under agarose, a few hours after WR infection, as previously observed in isolated infected cells in culture [31], [43]. This morphological change is dependent on F11, as well as its ability to bind RhoA, as it was not observed in ΔF11L or F11-VK infected cells. This suggests that virus induced cell rounding or contraction is at least in part dependent on the modulation of RhoA signalling as well as changes in cell adhesion and migration. Curiously, F11-VK infected cells still exhibited some loss of cell-cell adhesion and limited cell migration. These small changes cell-cell adhesion and cell migration, rather than viral release, probably accounts for the increased spread of F11-VK *in vivo* and larger plaques at 4 days post infection when compared to ΔF11L, as both viruses induce similar numbers of actin tails and also release similar amounts of infectious virus particles in the media.

The similar rate of ΔF11L or F11-VK spread observed during live cell imaging over the first 48 hours of infection would however, suggest that viral release rather than cell migration represents the main factor contributing to the intial spread of infection. This hypothesis also agrees with the delayed spread of F11-VK *in vivo* as compared to WR. Our data are also consistent with the notion that it is the extra-cellular enveloped virus (EEV), which is released from infected cells and resistant to complement or antibody mediated neutralization, that is largely responsible for the spread of infection throughout the mouse [44], [45], [46]. The residual RhoA binding observed in our F11-VK pull downs may account for the differences between ΔF11L and F11-VK viruses. However, the similar levels of GTP-bound RhoA in ΔF11L or F11-VK infected cells, would suggest that F11 has additional functions beyond inhibition of RhoA. One attractive possibility is that F11-VK interacts with additional RhoGTPases that are also involved in regulation of cell-cell adhesion and cell migration.

Finally, recombinant poxviruses vectors currently represent highly attractive delivery systems for vaccination against a variety of infectious diseases as well as oncolytic agents against a wide range of different cancers [47], [48], [49]. There is however still a need to improve the safety, immunogenicity and/or oncolytic activity of existing poxvirus based vectors if their full therapeutic potential in the clinic is to be realized. The reduced viral spread, mortality and the ability of mice to eliminate the ΔF11L virus from their lungs suggests that deletion of the F11L gene in existing vaccinia vectors may help to improve their safety and/or therapeutic potential as oncolytic agents by limiting the spread of infection.

## Materials and Methods

### Construction of ΔF11L and F11-VK Recombinant Viruses

The F12L gene was amplified by PCR from Western Reserve (WR) and cloned into the Not1/BamHI sites of pBS SKII containing the pE/L-gpt or the pE/L-gpt-pE/L-mCherry cassettes to generate F12L-pE/L-gpt and F12-pE/L-gpt-pE/L-mCherry. The last 386 bp of the F11L gene, which contains the promoter for F10L was cloned into the HindIII-SalI sites of F12L-pE/L-gpt and F12-pE/L-gpt-pE/L-mCherry vectors to generate the ΔF11L targeting vectors, F12L-pE/L-gpt-F11L (386 bp) and F12-pE/L-gpt-pE/L-mCherry-F11L (386 bp). F12-pE/L-gpt-pE/L-mCherry-F11L (386 bp) was transfected into cells infected with the ΔF12L virus [50]. Fluorescence and gpt were used as selectable markers to isolate a recombinant ΔF11L-mCherry virus by successive rounds of plaque purification. Subsequently, ΔF11L-mCherry virus infected cells were transfected with F12L-pE/L-gpt-F11L (386 bp) and the ΔF11L virus lacking mCherry was isolated by plaque purification. The F11-VK recombinant virus was isolated by rescuing the ΔF11L virus using targeting vectors in which the codons corresponding to valine 305 and lysine 307 of the F11L gene were changed to encode alanines. Cells infected with the ΔF11L and F11-VK viruses were subsequently transfected with the YFP-A3L targeting vector [11] and plaque purification was used to isolate the fluorescent viruses ΔF11L-YFP-A3L and F11-VK-YFP-A3L. The fidelity of all recombinant viruses was confirmed by sequencing.

### Infections, Immunofluorescence, and Antibodies

HeLa cells were infected and processed for immunofluorescence or immunoblot analysis as described previously [29] using the following antibodies: A27 (C3) [51], A36 [16], B5 (19C2) [52], F11 [28], F12 [10], GST polyclonal G-7781 (SIGMA-ALDRICH), GFP monoclonal 3E1 (CR-UK), Nck polyclonal 06-288 (Millipore) and RhoA monoclonal 26C4 (Santa Cruz Biotech, CA-USA).

### Quantification of Rho Activation, RhoA Pull Downs, and Virus Production

Rho activation assays were performed on lysates from HeLa cells infected for 8 hours as described previously [29]. The ratio of activated:total RhoA was determined in 4 independent experiments using Quantitative Westerns Methods on a Odyssey Infrared Imaging System (LI-COR Corporate, NE). The data for each virus is presented as the percentage of GTP bound Rho in non-infected control cells.

Vaccinia virus infected HeLa cells expressing GFP-Rho together with GST-F11 or GST were lyzed on ice in the Mg^2+^ Rho lysis buffer (Upstate) containing 25 mM NaF, 20 mM PMSF, 20 mM orthovanadate and protease inhibitors. Cell lysates were treated with 10 mM GDP or GTPγS for 30 minutes at 30°C after addition of 10 mM EDTA. Subsequently, the treated cell lysates were incubated with Glutathione sepharose 4B resin (Amersham) and processed as previously described [53].

The number of new infectious virus particles produced during infection was determined from triplicate plaque assays in four independent experiments. The data is presented as the percentage of the total infectious virus particles (intra-cellular, cell associated and released) produced during infection at 8 hours that are released into the media.

### Live Cell Imaging

Images from live BS-C-1 cells infected with WR, ΔF11L or F11L-VK viruses encoding YFP-A3L under agarose were collected every 10 minutes for 48 hours using a CP-Achromat 5x Objective (Carl Zeiss, Germany) and CoolSnap HQ camera (Photometrics, AZ) as described previously [29]. Quantification of plaque size over time was determined from the area of the YFP-A3L signal using MetaMorph (Molecular Devices Corporation, CA).

### Ethics Statement, Intranasal Inoculation, Virus Titration, and Statistical Analysis

The mouse experiments were approved by the Ethical Committee of Animal Experimentation (CEEA-CNB) of Centro Nacional de Biotecnologia (CNB-CSIC) in accordance with national and international guidelines and with the Royal Decree (RD 1201/2005). Animals were housed in ventilated racks and handled according to local and EU legal requirements. Two groups of six C57/BL6 mice were inoculated intranasally with 5×10^6^ plaque forming units of WR, ΔF11L or F11-VK viruses in 20 µl phosphate-buffered saline. Mice were monitored daily for survival. Inoculated animals were sacrificed at 1, 3 and 6 days post-inoculation, the organs removed, homogenized and the levels of infectious virus titrated as previously described [54]. Data are presented as mean±standard error of the mean and were analyzed by ANOVA or Student's t test using Prism 4.0 (GraphPad Software, CA). A P value of <0.05 was considered statistically significant.

## Supporting Information

Movie S1 The movie shows the formation of a representative plaque induced by Western Reserve virus encoding YFP-A3 over a period of 48 hours (time stamp indicates hours and minutes) after detection of the first infected cell. The right panel shows the signal of YFP-A3, a core viral protein, which highlights the spread of infection. The left panel phase image highlights the loss of cell-cell adhesion and the strong “contraction wave” at the advancing infection front.
(8.72 MB MOV)

Movie S2 The movie shows the formation of a representative plaque induced by the ΔF11L virus encoding YFP-A3L over a period of 48 hours (time stamp indicates hours and minutes) after detection of the first infected cell. The right panel shows the signal of YFP-A3, a core viral protein, which highlights the spread of infection. The left panel phase image reveals that ΔF11L infected cells do not detach from one another or undergo a strong “contraction wave” at the advancing infection front.
(7.28 MB MOV)

Movie S3 The movie shows the formation of a representative plaque induced the F11-VK virus encoding YFP-A3L over a period of 48 hours (time stamp indicates hours and minutes) after detection of the first infected cell. The right panel shows the signal of YFP-A3, a core viral protein, which highlights the spread of infection. The left panel phase image reveals that F11-VK infected cells, which do not contract, exhibit limited loss of cell-cell adhesion and viral-induced cell migration.
(6.51 MB MOV)

Movie S4 The movie shows a close-up taken from Movie S1 of the first 12 hours after detection of the first cell infected by Western Reserve virus encoding YFP-A3 at the start of plaque formation. The YFP-A3 signal highlights infected cells (right panel). The phase image shows that cells in the center of the plaque lose cell-cell adhesion, undergo contraction, and migrate away from the initial site of infection (left panel).
(1.54 MB MOV)

Movie S5 The movie shows a close-up taken from Movie S2 of the first 12 hours after detection of the first cell infected by the ΔF11L virus encoding YFP-A3 at the start of plaque formation. The YFP-A3 signal highlights infected cells (right panel). The phase image shows that ΔF11L-infected cells maintain cell-cell adhesion, and do not contact or migrate away from the initial site of infection (left panel).
(2.48 MB MOV)

Movie S6 The movie shows a close-up taken from Movie S3 of the first 12 hours after detection of the first cell infected by the F11-VK virus encoding YFP-A3 at the start of plaque formation. The YFP-A3 signal highlights infected cells (right panel). The phase image shows that F11-VK infected cells, while not contracting, exhibit limited loss of cell-cell adhesion and migration (left panel).
(2.22 MB MOV)
