# Correction: F11-Mediated Inhibition of RhoA Signalling Enhances the Spread of Vaccinia Virus In Vitro and In Vivo in an Intranasal Mouse Model of Infection

**DOI:** 10.1371/annotation/0c8d44be-4bff-49b3-855a-4cba76160a03

**Published:** 2010-06-08

**Authors:** João V. Cordeiro, Susana Guerra, Yoshiki Arakawa, Mark P. Dodding, Mariano Esteban, Michael Way

Susana Guerra and Michael Way contributed equally to this work.

